# Application of *Bacillus velezensis* NJAU-Z9 Enhanced Plant Growth Associated with Efficient Rhizospheric Colonization Monitored by qPCR with Primers Designed from the Whole Genome Sequence

**DOI:** 10.1007/s00284-018-1563-4

**Published:** 2018-09-07

**Authors:** Yang Zhang, Xu Gao, Shuai Wang, Chengzhi Zhu, Rong Li, Qirong Shen

**Affiliations:** 1 Jiangsu Provincial Key Lab for Organic Solid Waste Utilization, National Engineering Research Center for Organic-based Fertilizers, Jiangsu Collaborative Innovation Center for Solid Organic Waste Resource Utilization, Nanjing Agricultural University, Nanjing, 210095 China; 2 Ecology and Biodiversity Group, Department of Biology, Institute of Environmental Biology, Utrecht University, 3584 CH Utrecht, The Netherlands; 3 College of Resources and Environmental Sciences, Nanjing Agricultural University, Nanjing, 210095 China

## Abstract

Plant growth-promoting rhizobacteria (PGPR) have been reported to influence plant growth, yield, and nutrient uptake by an array of mechanisms. Uncovering the behavioral dynamics of PGPR is one of the most important issues necessary for understanding their functional performances. In this study, strain NJAU-Z9 which was found to possess complex functions and efficient rhizospheric colonization ability was selected from plenty of bacterial strains isolated randomly from the pepper rhizosphere soil and identified as *Bacillus velezensis*. Repeated seedling nursing tests performed absolute growth-promoting advantage for the novel isolated strain. After that, primers for the quantitative detection were designed based on its whole genome sequence (WGS), and a real-time PCR method was utilized to explore strategies for monitoring the strain in natural soil and in the pepper rhizosphere. Results showed based on the whole genome, two primers were identified as NJAU-Z9-specific quantitative PCR primers. Two seasonal pot experiments demonstrated that strain NJAU-Z9 effectively colonized the rhizosphere measured by the novel abundance detecting strategy, improved plant growth, and showed a positive correlation between bacterial number and biomass. This study offers a strategy based on a real-time PCR method for directly monitoring *B. velezensis* strain NJAU-Z9 in the soil and the rhizosphere and provides a reference for the quantitative study of other PGPR strains based on WGSs.

**Electronic supplementary material:**

The online version of this article (10.1007/s00284-018-1563-4) contains supplementary material, which is available to authorized users.

## Introduction

Plant growth-promoting rhizobacteria (PGPR) use one or more different mechanisms to promote plant growth and health directly or indirectly [1]. Among PGPR, *Bacillus* spp. possess excellent performance with many advantages, including wide distribution, easy isolation and culturing, and formation of oval endospores [2] Many representatives of this genus perform functions such as the solubilization of nutrients, nitrogen fixation, and the production of growth regulators and are related to the growth promotion efficiency observed in inoculated plants [3]. In the past dozen years, in order to have beneficial effects on a target plant, numerous studies have introduced PGPR agents in large number by seed or seed piece inoculation and soil amendment [4, 5]. Survival of these inoculated strains in a new environment, however, does play a critical role in function performance, since effective colonization is necessary for the successful stimulation of plant growth or the soil microbial ecosystem by the target strain [6]. Thus, one of the most important issues in the field is to uncover the behavioral dynamics of the inoculants in the soil.

Early studies focused on culturing strategies to monitor functional microbes [7]; however, single cell and culture conditions do not adequately replicate the natural environment [8]. Moreover, species/strain-specific selective media are seldom available; the procedure is extremely time consuming; and the results are not immediate. In fact, agricultural soils are rich in a wide variety of native microorganisms, and most species in many environments have never been described [9, 10]. Contemporarily, based on DNA analyses, a variety of molecular methods are employed to identify the abundance of functional microbes as well as different genes that could be involved in soil processes [11, 12]. Among these, real-time quantitative PCR (qPCR) is widely used not only for the detection and quantification of 16S rRNA genes [13] but also for identifying functional genes involved in rhizosphere-related processes due to its high specificity, sensitivity, and speed [14]. Although these reports showed that qPCR is a valuable technique for quantitatively monitoring populations of unlabeled bacteria in greenhouse experiments, utilization of strain primers leads to robust detection for target microbes. In addition, the application of strain primers is more difficult in field experiments where closely related indigenous bacteria may interfere with amplification and quantification.

Since a type strain CBMB205^T^ (= KACC 13015^T^), recognized as separate from other members of the genus *Bacillus*, was isolated from rice rhizosphere soil (Korea, in 2010), novel species named *Bacillus velezensis* was suggested [15]. Actually, only minor differences of the genomes between this strain and *B. velezensis* NRRL B-41580^T^, *B. oryzicola* KACC 18228, and *Bacillus amyloliquefaciens* subsp. *plantarum* FZB42^T^ based on comparative genomic analysis were observed, and *B. methylotrophicus, B. amyloliquefaciens* subsp. *plantarum*, and *B. oryzicola* were later proposed to be heterotypic synonyms of *B. velezensis* [15–20]. However, regardless of the taxonomy of *B. velezensis*, these strains were commonly isolated as plant pathogen antagonists and developed as biological control and plant growth promotion agents [21, 22]. Nevertheless, few studies have focused on the quantification of the *B. velezensis* group.

In this study, a promising phytostimulatory plant growth-promoting bacterium (PGPR), NJAU-Z9, isolated from pepper rhizosphere was identified as *B. velezensis*, and it showed a significant promoting effect on pepper growth by pre-colonization at the seedling stage. The whole genome was sequenced by Single-Molecule, Real-Time (SMRT) technology, and the unique and efficient quantitative primers were designed based on genome fragments to explore strategies for monitoring the functional species using qPCR (Fig. S1) and subsequently to verify the correlation between the number of strain NJAU-Z9 in the rhizosphere and plant growth promotion (Fig. S2). It is hoped that the results will provide technical assistance to microbial ecology researchers of the species in the future and will be a reference for the quantitative study of other strains based on whole genome sequences (WGSs).

## Materials and Methods

### Bacterial Isolation

Bacteria were obtained from a collection of microbes originally isolated from a pepper rhizosphere grown in China (31°43′N, 118°46′E). The preliminary screening process is described in Zhang et al. [23]. A potential carbon source utilization of the isolates was assessed by using the BIOLOG(R) GEN III MicroPlate (Biolog Inc., Hayward, CA) according to manufacturer’s instructions. Production of IAA-like auxins, ammonia production, and an in vitro antagonism assay were performed according to references [24]. One bacterial strain NJAU-Z9 was identified based on its morphological, physiological, and biochemical properties according to Bergey’s Manual of Determinative Bacteriology. The 16S rRNA-related taxa of strain NJAU-Z9 and the sequence similarity were analyzed using a global alignment algorithm, implemented at the EzTaxon-e server [25]. A phylogenetic tree of 16S rRNA gene sequences was reconstructed using the maximum-likelihood (ML) [26] algorithms in the MEGA version 7.0 software. Bootstrap values were set as 1000 replications for analysis. Average nucleotide identity (ANI) values of the closest strains were estimated using the algorithm developed by Goris et al. [27], as implemented in the server EzBioCloud available at http://www.ezbiocloud.net/tools/ani [28]. The WGS of closest type strains were downloaded from the GenBank DNA database and have been described in Table S1.

### Whole Genome Sequence Analyses

The sequencing of the NJAU-Z9 genome completion map was performed at Novogene Biotechnology Co, Ltd. (Beijing, China) by Single-Molecule, Real-Time (SMRT) technology [29]. The complete genome of *B. velezensis* NJAU-Z9 is publicly available in the NCBI database under accession number NZ_CP022556.1. It contains a 3,847,297 bp circular chromosome with a GC content of 46.78%, a 17,243 bp circular plasmid-1 with a GC content of 39.52%, and an 8020 bp non-circular plasmid-2 with a GC content of 39.98%. In total, 55,930 reads with 195.78-fold coverage were produced, and there are 3983 predicted genes in the chromosome, including 86 tRNA-encoding genes and 27 rRNA-encoding (5S, 16S, and 23S) genes.

### Primer Design, Evaluation, and Target Plasmid Construction

The coding sequence (CDS) from the NJAU-Z9 whole genome was used for primer design. All fragments of the CDS were subjected to a BLASTn search one by one against the NCBI database (GenBank release 214), and 5 fragments without any matching information were obtained. Among them, there are three segments larger than 1000 bp and two segments smaller than 200 bp. Two small fragments were discarded and three large fragments were used to design sets of primer pairs for NJAU-Z9 (positions 3017742–3018779, 3019264–3020556, and 3020642–3022177). The designed primer pairs were analyzed using Oligo 6, synthesized by Nanjing GenScript Biotechnology Co., Ltd. (China) and qualitatively detected by conventional PCR. Eight-pair primers were used as candidates, the optimal amplification conditions for each pair are shown in Table 1 (Fig. S1 describes the design process in detail), and the primer pair *Bacillus* was used as a positive amplification control [30]. The nine primer pairs were tested for cross amplification against DNA sequences from six other *Bacillus* species and our target strain as templates (Table S3). Different amplification products were generated by conventional PCR from NJAU-Z9 genomic DNA using the eight primer pairs and subsequently cloned into pMD19-T vectors (TaKaRa). The recombinant plasmids were transformed into *E. coli* DH5a and extracted and purified with the AxyPrep Plasmid Miniprep Kit (Axygen Inc., USA).

**Table 1:** Primer characteristics and parameters evaluated by qPCR

Genome location or reference	Code	Primer set	Sequence (5′–3′)	Fragment size (bp)	Optimum conditions	R^2^	Slope	Efficiency (%)
Chr1:3019264:3020556		Z9-F	AGACTTATTCGCATTTAGGC	76	1 min incubation at 95 °C, 40 cycles consisting of 95 °C for 15 s and 60 °C for 34 s	0.992	− 3.325	96.4
	P8	Z9-R	ATCTTCTGTGCTCTTTCGAC					
		Z9-1F	ATGCGTCTCATATGGATAGA	215	1 min incubation at 95 °C, 40 cycles consisting of 95 °C for 15 s, and 60 °C for 30 s followed by 72 °C for 30 s	0.993	− 3.429	95.7
	P1	Z9-1R	ATTAGTAAAGTCAGCAACAA					
		Z9-2F	GACAAGCTAAATAACCACTA	205	1 min incubation at 95 °C, 40 cycles consisting of 95 °C for 15 s and 60 °C for 30 s followed by 72 °C for 30 s	0.995	− 3.436	102.8
	P2	Z9-2R	TAAGATACCTATGATCCAAT					
Chr1:3020642:3022177		Z9-3F	GTGGTAGTAACGCTATTGAA	143	1 min incubation at 95 °C, 40 cycles consisting of 95 °C for 15 s and 60 °C for 30 s followed by 72 °C for 30 s	0.993	− 3.409	96.5
	P3	Z9-3R	CCTCTTTACCCACAACTGCA					
		Z9-4F	GTGGTAGTAACGCTATTGAA	147	1 min incubation at 95 °C, 40 cycles consisting of 95 °C for 15 s and 62 °C for 34 s	0.997	− 3.329	99.7
	P4	Z9-4R	AAGTAACCTCTTTACCCACA					
Chr1:3017742:3018779		Z9-5F	GGCTCGTGGCACAATAGACC	280	1 min incubation at 95 °C, 40 cycles consisting of 95 °C for 15 s and 62 °C for 34 s	0.996	− 3.388	97.3
	P5	Z9-5R	AGCGTGTTTAGGTGCTTTGA					
		Z9-6F	AGGATACAACGATTCTTACA	123	1 min incubation at 95 °C, 40 cycles consisting of 95 °C for 15 s and 62 °C for 34 s	0.994	− 3.393	97.1
	P6	Z9-6R	CACTTTGGAATCTGGTCTAT					
		Z9-7F	TATCAAAGCACCTAAACACG	162	1 min incubation at 95 °C, 40 cycles consisting of 95 °C for 15 s and 60 °C for 30 s followed by 72 °C for 30 s	0.998	− 3.248	103.2
	P7	Z9-7R	AGATTATCGGCGGTAGCAAA					
[30]	Primer *Bacillus*							

### DNA Extraction and Real-Time Quantitative PCR

Genomic DNA of NJAU-Z9 and soil DNA were extracted using the Bacterial DNA kit (Omega) and PowerSoil DNA Isolation Kit (Mo Bio Laboratories, Carlsbad, CA, USA) following the manufacturer’s instructions. The qPCR amplification and detection were performed in a 20 µl reaction volume. The cycle threshold (CT) value was automatically determined for each sample with the ABI Prism 7500 system. After amplification, the melting curve and amplification efficiency analysis were performed to confirm amplification specificity. The Ct values were determined for all assays, and initial target gene copy numbers in unknown samples were calculated from the standard curves (all gene fragments are single copy). The plate counting was according to Zhang et al. [23]. The natural soil samples’ colony counts were expressed as the number of CFU per gram (dry weight) of soil, and qPCR quantification data were converted to the equivalent number of CFU per gram (dry weight) of soil. In the pot experiment, total numbers of NJAU-Z9 were quantified by qPCR with primers P4, which was selected by the previous work of this experiment. Each sample was performed in 12 replicates, and the results were expressed as log (CFU g^−1^) dry soil.

### Natural Soil Samples, Seeding, and Pot Experimental Layout

Natural soil samples of 10 g were amended in 24 50-ml centrifuge tubes, and half of them were sterilized at 121 °C for 20 min. The NJAU-Z9 cells prepared according to Zhang et al. [31] were inoculated in six sterile and six non-sterile tubes with approximately 10^7^ CFU g^−1^ soil each and divided into two groups. One of the sterile tubes was amended with cells and detected immediately, another was detected 5 days later, and a control with no inoculation was prepared. The other group contained unsterilized soil amended with cells and detected immediately, unsterilized soil amended with cells detected 5 days later, and a control with no inoculation. All treatments were performed in triplicate (Fig. S2a described the design process in detail). Plant growth-promoting efficiency of strain NJAU-Z9 was evaluated by seedlings nursing and pot experiments in a greenhouse, and the test pepper variety is sweet pepper (*Capsicum annuum* L.). The seedlings nursing experiment contained one treatment and a control using ordinary nursery substrate amended with or without strain NJAU-Z9 (5% v/w dry weight). Treatment and control were designed with three replicates. Each replicate included eight seedlings. Pepper seedlings were cultivated in the substrates for 40 days, and the agronomic characteristics and biomasses were measured. After that, pot experiments were further performed to validate the plant growth promotion effects of the seedlings cultured from nursery substrate with strain NJAU-Z9. Pepper seedlings were transferred into pots with 5 kg soil and supplemented with 1.5% (w/w dry weight) chicken manure compost. The seedlings of the treatment and control groups with 25 replications were prepared by the nursery substrate produced by strain NJAU-Z9 (OFBS) and ordinary substrates (OF), respectively. All pots were placed within a randomized block design. At the end of the experiment, the agronomic characteristics and biomasses of the plants were measured. Pot experiments was repeated twice. The qPCR assay was performed to detect the number of target strain in pepper. The first pot experiment was conducted to verify the ability of NJAU-Z9 to stabilize in rhizosphere throughout the growth period, and in the second pot experiment, the amount was monitored at the mature period (Fig. S2b described the design process in detail).

### Data Analysis

Statistical analysis was performed by using the IBM SPSS 18.0 software program (IBM Corporation, New York, USA). All statistical tests performed in this study were considered significant at *P* < 0.05. The data were subject to the Student *t* test (to compare means of two treatments) and to analysis of variance (to compare many treatments) with means compared by the Tukey test. Colony counts were expressed as the number of CFU per gram (dry weight), and qPCR quantification data were converted to the equivalent number of CFU per gram (dry weight). Pearson correlation coefficients between the qPCR quantification data of NJAU-Z9 and plant growth index were calculated in R software (Version 3.3.3).

## Results

### Isolation and Identification of Strain PGPR NJAU-Z9

**Fig. 1:**
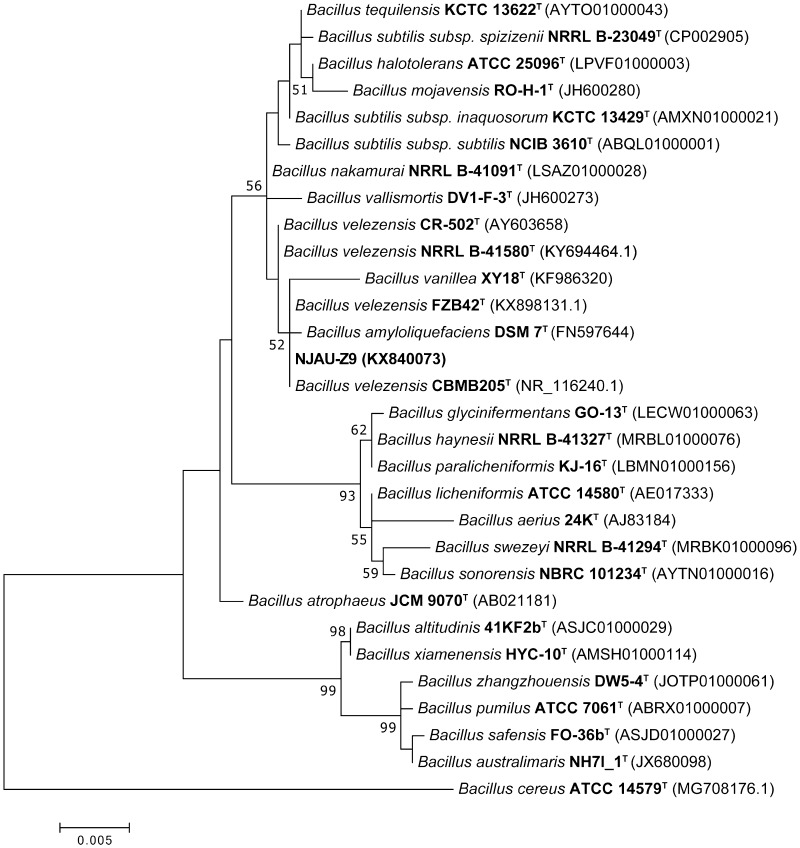
Maximum-likelihood phylogenetic tree based on the 16S rRNA gene sequences of strain NJAU-Z9 and related strains in the genus *Bacillus*. Bootstrap values are shown as percentages of 1000 replicates; values below 50% are not indicated. *Bacillus cereus* ATCC 14579^T^ were used as outgroups. Accession numbers are given in parentheses. Bar, 0.005 substitutions per nucleotide position

Approximately 150 bacterial strains were isolated from the rhizospheric soil of sweet pepper (*Capsicum annuum* L.). One strain that was the best at both producing indole acetic acid and NH_3_ simultaneously and acting in antagonism against *Fusarium oxysporum f.sp. niveum* and *Ralstonia solanacearum* (data not shown) was named NJAU-Z9. Strain NJAU-Z9 is a rod-shaped, Gram-positive bacterium with peritrichous flagella and able to form oval endospores. Characteristics detected by BIOLOG GEN III MicroPlate are shown in Table S2. Strain NJAU-Z9 was automatically classified into *Bacillus* sp. The 16S rRNA gene sequence similarity calculations indicated that strain NJAU-Z9 was closely related to the species of the genus *Bacillus* and shared the highest sequence similarity with *B. velezensis* CBMB205^T^ (100%), and *B. velezensis* FZB42^T^ (100%), followed by *B. amyloliquefaciens* DSM 7^T^ (99.92%) and *B. velezensis* CR-502^T^ (99.92%). Phylogenetic analysis based on 16S rRNA gene sequences showed that strain NJAU-Z9 belonged to the genus *Bacillus* and formed a subclade with *B. velezensis* CBMB205^T^ and *B. velezensis* FZB42^T^ (Fig. 1) with a robust bootstrap support (value 100%) in the maximum-likelihood tree. In addition, the ANI values between NJAU-Z9 and the type strains *B. velezensis* CBMB205^T^, *B. velezensis* FZB42^T^, and *B. amyloliquefaciens* DSM 7^T^ are all greater than 94%, and *B. velezensis* CBMB205^T^ is the closest one with ANI value of 97.94%. Moreover, these strains all have highly similar GC contents to NJAU-Z9 (Table S1). Thus, on the basis of morphological, physiological, and biochemical properties combined with 16S rRNA and WGSs analysis, strain NJAU-Z9 was finally identified as *B. velezensis*.

### Primer Design, Evaluation, and Amplification Efficiency

**Fig. 2:**
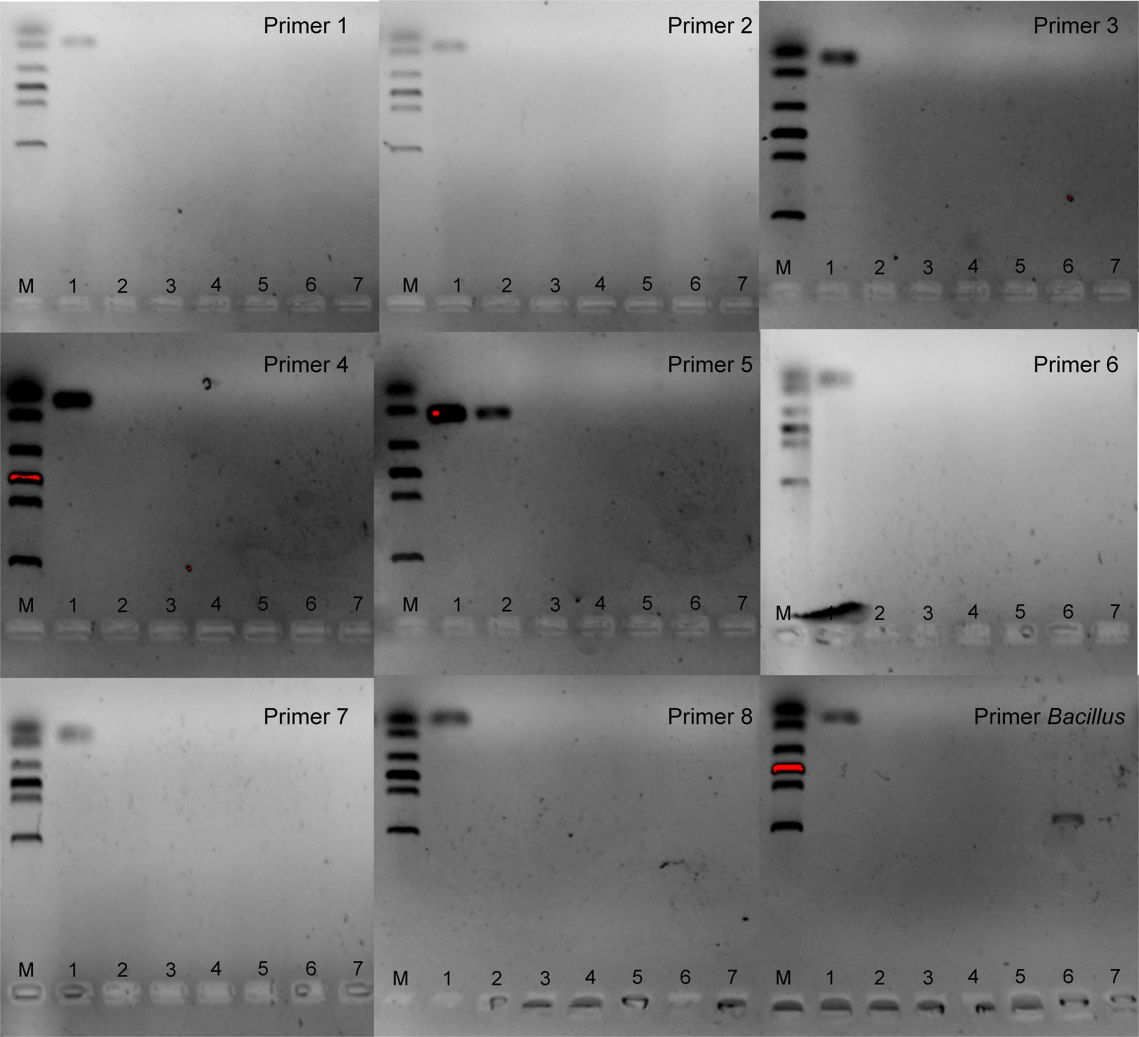
Specificities of the primer pairs designed to amplify different *Bacillus* species. Lane M, DL2000 DNA Marker; Lane 1, strain NJAU-Z9; 2, *B. subtilis* NJAU-G10; 3, *B. amyloliquefaciens* SQR-9; 4, *B. amyloliquefaciens* T-5; 5, *B. pumilus* LZ-8; 6, *B. amyloliquefaciens* NJN-6; and 7, *B. vallismortis* NJAU-N23. Primers 1 to 8 were designed from the genome of *B. velezensis* NJAU-Z9; Primer *Bacillus* was used as a positive amplification control

Three coding and intergenic regions from the NJAU-Z9 genome were selected, and a total of 8 primer pairs were designed and tested. Unique amplicons of expected size were observed on agarose gel electrophoresis (Fig. S3b) for each primer with no amplification with any of miscellaneous bands, and the melting point temperatures produced by these primers were approximately 76–83 °C with a single melting peak shape (Fig. S3a). Primer pairs P1, P2, P3, P4, P6, P7, and P8 produced amplicons only when *B. velezensis* NJAU-Z9 DNA was used as the template and were considered strain primer pairs; primer *Bacillus* was not able to amplify all *Bacillus* strains tested and primer pair P5 produced cross-species amplicons (amplification for *B. subtilis* strain G10 was observed), which was discarded from further analyses (Fig. 2). In addition, amplification of all *Bacillus* strains using universal primers for the 16S rRNA gene was used as another positive control, however, as expected, 16S rRNA gene universal primers (27F 5′-AGAGTTTGATCCTGGCTCAG-3′ and 1492R 5′-GGTTACCTTGTTACGACTT-3′) was able to amplify all *Bacillus* strains tested (Fig. S4).

### Plant Growth Promotion Effect

Except for the SPAD and leaf number, the other agronomic traits of the pepper seedlings growing in the nursery substrate amended with strain NJAU-Z9 were significantly higher than those in the control in all three replicates of the seedling cultivation experiments. These results indicate that the functional strain NJAU-Z9 is beneficial for improving pepper seedling growth (Table 2 and Fig. S5). In the second season of the pot experiments, the plant height and stem diameter of the pepper trees were greatly enhanced in the treatment (OFBS) inoculated strain NJAU-Z9, especially for the yields, which were significantly increased (*P* < 0.05) by 36.9% and 32.8%, respectively, when compared to the OF (Table 3 and Fig. S5).

**Table 2:** Effects of the inoculation of nursery substance with strain NJAU-Z9 on pepper seedling growth

Treatments	Plant height (cm)	Stem diameter (mm)	Leaf size (cm^2^)	SPAD	Leaf number
Experiment 1					
NJAU-Z9	9.17 ± 0.41*	1.78 ± 0.07*	5.02 ± 0.12*	44.4 ± 0.89	5
CK	8.12 ± 0.24	1.68 ± 0.02	4.34 ± 0.09	43.57 ± 0.12	4
Experiment 2					
NJAU-Z9	13.06 ± 0.37*	2.33 ± 0.01*	13.19 ± 0.22*	43.63 ± 0.84	5
CK	12.11 ± 0.2	2.19 ± 0.02	11.61 ± 0.23	42.97 ± 0.34	5
Experiment 3					
NJAU-Z9	10.02 ± 0.22*	1.76 ± 0.03*	6.1 ± 0.05*	41.37 ± 0.52*	4
CK	9.15 ± 0.38	1.41 ± 0.05	5.27 ± 0.14	39.13 ± 0.11	4

Values are means ± standard deviation

*Represent significant difference (*t* test, *P* < 0.05)

**Table 3:** Agronomic traits of pepper after transplanting for 40 days in the pot experiment

Treatments	Plant height (cm)	Stem diameter (mm)	Biomass (g)	SPAD
Experiment 1				
NJAU-Z9	51.67 ± 1.76*	6.35 ± 0.82	1282.38 ± 41.89*	46.4 ± 0.54
CK	45.33 ± 4.68	5.68 ± 0.33	936.46 ± 62.41	47.57 ± 0.69
Experiment 2				
NJAU-Z9	45.26 ± 6.89*	5.33 ± 0.41*	972.45 ± 43.21*	43.28 ± 1.24
CK	38.42 ± 2.66	4.19 ± 0.22	731.83 ± 27.69	44.51 ± 0.33

Values are means ± standard deviation

*Represent significant difference (*t* test, *P* < 0.05)

### Quantification of NJAU-Z9

**Fig. 3:**
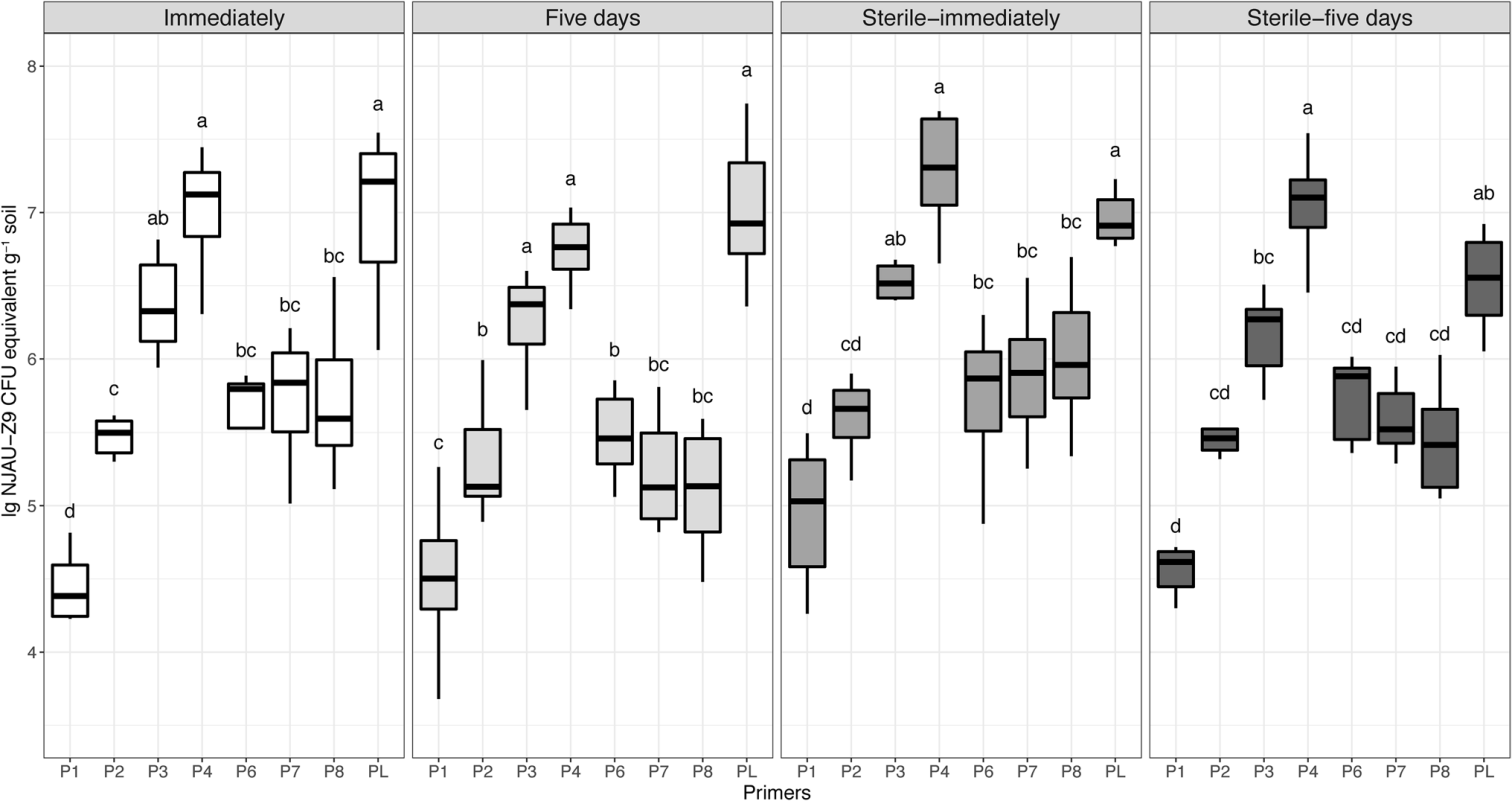
Detection of strain NJAU-Z9 by qPCR and plate counting (PL) methods in sterile and non-sterile soils. The values for qPCR are the means from three experiments using different strain primers. Sterile-immediately: immediately detected under sterile soil; sterile-five days: detection was performed after 5 days under sterile soil; immediately: immediately detected under non-sterile soil; 5 days: detection was performed after 5 days under non-sterile soil. The bars are the respective standard deviations (*n* = 12). The letters indicate significant differences among primers determined by the Tukey test

As shown in Fig. 3, the amounts of NJAU-Z9 in all sterile soil samples were slightly higher than that in the non-sterile soils regardless of measured on the first day (immediately) or the fifth day, while in non-sterile soils, the amounts of NJAU-Z9 in the fifth day were slightly lower than that on the first day. Moreover, among all primer pairs, the amounts from P1 were lower than others, the values of P4 were significantly higher than those of P2, P6, P7, and P8, and no significant difference was observed between P4 and P3 except on the fifth day in the sterile soil. In addition, the amounts from P3 and P4 showed no significant difference with plate counts (PL) (Fig. 3). Since the target gene fragments of all primers are single copies, based on the plate counts data as a reference, it can be concluded that the detection values from P3 and P4 were close to the true values (approximately 10^7^ CFU g^−1^ soil). Moreover, soil samples from the sterile-control and control groups (immediately and five days, respectively) were also analyzed by using qPCR and plate counts method, and the CT values were both above 32, with no strains observed on the plates, indicating that the original soil did not contain the target strain (data not shown).

**Fig. 4:**
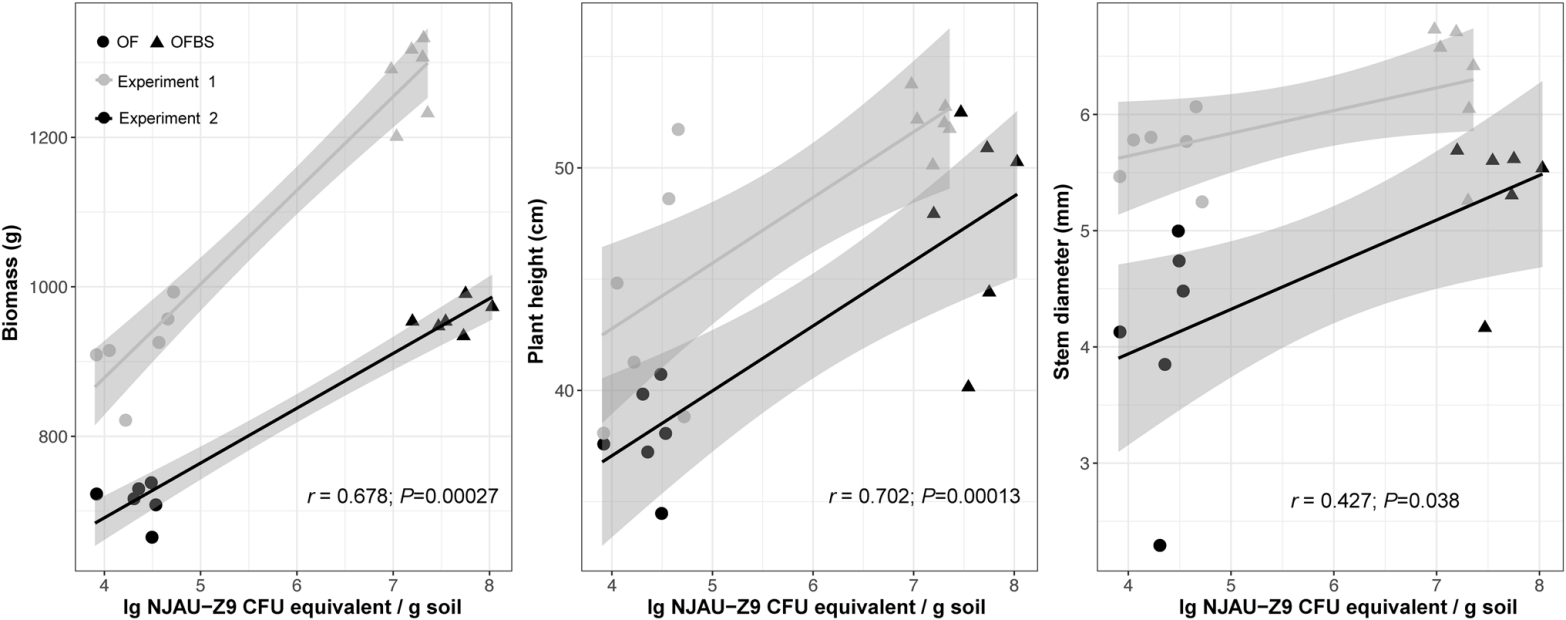
Pearson correlations (*r*) between the qPCR quantification data of strain NJAU-Z9 and plant growth index of mature period in two pot experiments

In two seasons of the pot experiments, the number of strain NJAU-Z9 was effectively detected in the OFBS treatment-transplanted seedlings cultivated in the nursing substrates and amended with strain NJAU-Z9. In the first pot experiment, the number of strain NJAU-Z9 exhibited a stable rhizosphere colonization during the entire growing period of pepper (Fig. S6). The number was significantly higher (*P* < 0.05) than that detected in the OF control regardless of the different stages in the first or second pot experiment. Most importantly, Pearson correlation coefficients were also calculated between the number of strain NJAU-Z9 at the mature stage in two pot experiments and the plant growth index significantly positively correlated with biomass (*r* = 0.678, *P* = 0.000027), plant height (*r* = 0.702, *P* = 0.00013), and stem diameter (*r* = 0.427, *P* = 0.038) (Fig. 4).

## Discussion

In accordance with our results that pepper seeds inoculated with *B. velezensis* strain NJAU-Z9 obviously grew better than the control, all plant parameters showed enhancement when *Cajanus cajan* seeds were inoculated with functional microbes [32], and an increase of biomass after inoculation with *B. phytofirmans* PsJN has been reported in maize, wheat, and *Acacia ampliceps* [33, 34].

To date, a certain amount of microbial species such as *Paecilomyces lilacinus, Lactobacillus*, and *Enterobacter radicincitans*, were successfully monitored in various environments by qPCR using species-specific PCR primers [14, 35–37]. In order to solve the quantitative and dynamic monitoring of inoculated strains, we developed a strain qPCR protocol based on whole genome analysis to quantify *B. velezensis* strain NJAU-Z9. Eight-pair primers were designed by in silico comparison of the fragments from the WGS of *B. velezensis* NJAU-Z9, and the optimum amplification conditions were obtained by analyzing their dissolution curves and amplification efficiencies. Similarly, different methods, usually based on different experimental approaches, were used to design taxon-specific primers. Briefly, sequence-characterized amplified region (SCAR) markers obtained from BOX-PCR, enterobacterial repetitive intergenic consensus sequence-PCR, and randomly amplified polymorphic DNA (RAPD)-PCR fragments were recently applied to design primers for the qPCR quantification of *Azospirillum brasilense* and *Azospirillum lipoferum* at the strain level [38, 39]. However, these methods are more time consuming and insensitive compared to the method based on the WGS of the target species, which has been proven to be a rapid and reliable approach to provide comprehensive information about an organism and to detect specific DNA regions to be used as strain genetic markers for the quantitative detection of bacterial strains [40]. Primer verification assays have been performed using genomic DNA sequences from six other *Bacillus* species and our target strain as templates, and except primers P5, the others are strain specific which cannot amply any gene segment. In accordance with our results, four primer pairs were designed from *Azospirillum brasilense* FP2 whole genome, and after verification assays, three were left as strain primer pairs [41]. Thus, WGS offers genetic information with the highest resolution to explore novel strategies for monitoring the target microbes.

Results from natural soil using qPCR based on the strain primers showed that P3 and P4 primers were identified as NJAU-Z9-specific quantitative PCR primers in combination with the plate counts. Similar to our results, Stets et al. [41] also used the plate count data as a reference for determining primer pairs when monitoring *Azospirillum brasilense* FP2 on wheat roots in sterile and non-sterile conditions. For bacteria, there is a similar relationship between the number of single-copy genes and the number of colonies (CFU), and in the short-term inoculation experiment, the use of plate count data is a powerful reference for the effectiveness of specific primers [42]. Besides, in ecological research, the soil biodiversity of non-sterile soils is significantly different from that of sterile soils [43], our results also showed that the amount of *B. velezensis* NJAU-Z9 in sterile soil is slightly higher than that in non-sterile soil.

Pot experiments results showed that the inoculation of *B. velezensis* NJAU-Z9 significantly promoted plant growth. Similar to our results, strain *B. velezensis* NKG-1 was isolated and reported to suppress mycelial growth and conidial germination and to promote tomato plant growth [44]. Additionally, strain *B. velezensis* RC218 was reported to reduce Fusarium head blight and deoxynivalenol accumulation [45]. During the pot experiments, *B. velezensis* strain NJAU-Z9 stably colonized the rhizosphere, and the number present at the mature stage was positively correlated with plant growth. The results were consistent with common growth-promoting effects among numerous PGPR in that a gradual increase in equivalent cell numbers of PGPR detected by qPCR was observed over time, along with a simultaneous increase in plant growth promotion [46].

In conclusion, inoculation of *B. velezensis* strain NJAU-Z9 in the ordinary nursery substrate enhanced the plant growth, regardless in the seed nursing and subsequently transplanting stages. Two primer pairs from WGS of *B. velezensis* strain NJAU-Z9 were successfully designed and tested to monitor the fluctuation in the population of this strain by qPCR after inoculation into soil samples under sterile and non-sterile conditions. The detection efficiency was also successfully verified in two seasonal pot experiments, and the results demonstrated that *B. velezensis* strain NJAU-Z9 maintained high numbers during different growing stages, which were positively correlated with plant growth. In summary, the strategy for the design of strain primers described here may theoretically be used for different microbes for which the WGS is available in a database, and a qPCR method with strain primer pairs can be effectively applied to quantitatively monitor the population of PGPR in different environments.

## Electronic supplementary material

Below is the link to the electronic supplementary material.

Supplementary material 1 (TIF 2174 KB)

Supplementary material 2 (TIF 1061 KB)

Supplementary material 3 (TIF 1497 KB)

Supplementary material 4 (TIF 795 KB)

Supplementary material 5 (TIF 2099 KB)

Supplementary material 6 (TIF 1074 KB)

Supplementary material 7 (DOCX 25 KB)

## References

[1] Kloepper JW, Schroth MN (1978) Plant growth-promoting rhizobacteria on radishes. In: Proceedings of the 4th international conference on plant pathogenic bacteria. Tours, pp 879–882

[2] Collins DP, Jacobsen BJ (2003). Optimizing a *Bacillus subtilis* isolate for biological control of sugar beet cercospora leaf spot. Biol Control.

[3] Lópezbucio J, Camposcuevas JC, Hernándezcalderón E, Velásquezbecerra C, Faríasrodríguez R, Macíasrodríguez LI, Valenciacantero E (2007). *Bacillus megaterium* rhizobacteria promote growth and alter root-system architecture through an auxin- and ethylene-independent signaling mechanism in *Arabidopsis thaliana*. Mol Plant Microbe Interact.

[4] Ramos B, GarcíA JAL, Probanza AN, Barrientos ML, Mañero FJG (2003). Alterations in the rhizobacterial community associated with European alder growth when inoculated with PGPR strain *Bacillus icheniformis*. Environ Exp Bot.

[5] Ramamoorthy V, Viswanathan R, Raguchander T, Prakasam V, Samiyappan R (2001). Induction of systemic resistance by plant growth promoting rhizobacteria in crop plants against pests and diseases. Crop Prot.

[6] Dobbelaere S, Croonenborghs A, Thys A, Ptacek D, Okon Y, Vanderleyden J (2002). Effect of inoculation with wild type *Azospirillum brasilense* and *A. irakense* strains on development and nitrogen uptake of spring wheat and grain maize. Biol Fertil Soils.

[7] Bashan Y, Gonzalez LE (1999). Long-term survival of the plant-growth-promoting bacteria *Azospirillum brasilense* and *Pseudomonas fluorescens* in dry alginate inoculant. Appl Microbiol Biotechnol.

[8] Ritz K (2007). The Plate debate: cultivable communities have no utility in contemporary environmental microbial ecology. FEMS Microbiol Ecol.

[9] Streit WR, Schmitz RA (2004). Metagenomics-the key to the uncultured microbes. Curr Opin Microbiol.

[10] Amann RI, Ludwig W, Schleifer KH (1995). Phylogenetic identification and in situ detection of individual microbial cells without cultivation. Microbiol Rev.

[11] Zelles L, Bai Q, Beck T, Beese F (1992). Signature fatty acids in phospholipids and lipopolysaccharides as indicators of microbial biomass and community structure in agricultural soils. Soil Biol Biochem.

[12] Gattinger A, Ruser R, Schloter M, Munch JC (2002). Microbial community structure varies in different soil zones of a potato field. J Plant Nutr Soil Sci.

[13] Rosado A, Seldin L, Wolters A, Van Elsas J (1996). Quantitative 16S rDNA-targeted polymerase chain reaction and oligonucleotide hybridization for the detection of *Paenibacillus azotofixans* in soil and the wheat rhizosphere. Fems Microbiol Ecol.

[14] Nannipieri P, Ascher J, Ceccherini MT, Landi L, Pietramellara G, Valori F, Nautiyal CS, Dion P (2008). Effects of root exudates in microbial diversity and activity in rhizosphere soils. Molecular mechanisms of plant and microbe coexistence.

[15] Madhaiyan M, Poonguzhali S, Soonwo K, Tongmin S (2010). *Bacillus methylotrophicus* sp. nov., a methanol-utilizing, plant-growth-promoting bacterium isolated from rice rhizosphere soil. Int J Syst Evol Microbiol.

[16] Ruizgarcía C, Béjar V, Martínezcheca F, Llamas I, Quesada E (2005). *Bacillus velezensis* sp. nov., a surfactant-producing bacterium isolated from the river Velez in Malaga, southern Spain. Int J Syst Evol Microbiol.

[17] Borriss R, Chen XH, Rückert C, Blom J, Becker A, Baumgarth B (2011). Relationship of clades associated with strains DSM 7 and FZB42: a proposal for nov. and nov. based on their discriminating complete genome sequences. Int J Nurs Stud.

[18] Ma L, Cao YH, Cheng MH, Huang Y, Mo MH, Wang Y, Yang JZ, Yang FX (2013). Phylogenetic diversity of bacterial endophytes of *Panax notoginseng* with antagonistic characteristics towards pathogens of root-rot disease complex. Antonie Van Leeuwenhoek.

[19] Chung EJ, Hossain MT, Khan A, Kim KH, Che OJ, Chung YR (2015). *Bacillus oryzicola* sp. nov., an endophytic bacterium isolated from the roots of rice with antimicrobial, plant growth promoting, and systemic resistance inducing activities in rice. Plant Pathol J.

[20] Dunlap CA, Kim SJ, Kwon SW, Rooney AP (2016). *Bacillus velezensis* is not a later heterotypic synonym of *Bacillus amyloliquefaciens; Bacillus methylotrophicus, Bacillus amyloliquefaciens* subsp. *plantarum* and *‘Bacillus oryzicola*’ are later heterotypic synonyms of *Bacillus velezensis* based on phylogenomics. Int J Syst Evol Microbiol.

[21] Idris ESE, Iglesias DJ, Talon M, Borriss R (2007). Tryptophan-dependent production of indole-3-acetic acid (IAA) affects level of plant growth promotion by *Bacillus amyloliquefaciens* FZB42. Mol Plant Microbe Interact.

[22] Wu B, Wang X, Yang L, Yang H, Zeng H, Qiu Y, Wang C, Yu J, Li J, Xu D (2016). Effects of *Bacillus amyloliquefaciens* ZM9 on bacterial wilt and rhizosphere microbial communities of tobacco. Appl Soil Ecol.

[23] Zhang Y, Wen CY, Zhao MQ, Zhang M, Gao Q, Li R, Shen QR (2015). Isolation of plant growth promoting rhizobacteria from pepper and development of bio-nursery substrates. J Nanjing Agric Univ.

[24] Glickmann E, Dessaux Y (1995). A critical examination of the specificity of the salkowski reagent for indolic compounds produced by phytopathogenic bacteria. Appl Environ Microbiol.

[25] Kim OS, Cho YJ, Lee K, Yoon SH, Kim M, Na H, Park SC, Jeon YS, Lee JH, Yi H (2012). Introducing EzTaxon-e: a prokaryotic 16S rRNA gene sequence database with phylotypes that represent uncultured species. Int J Syst Evol Microbiol.

[26] Felsenstein J (1981). Evolutionary trees from DNA sequences: a maximum likelihood approach. J Mol Evol.

[27] Goris J, Konstantinidis KT, Klappenbach JA, Coenye T, Vandamme P, Tiedje JM (2007). DNA–DNA hybridization values and their relationship to whole-genome sequence similarities. Int J Syst Evol Microbiol.

[28] Yoon SH, Ha SM, Lim J, Kwon S, Chun J (2017). A large-scale evaluation of algorithms to calculate average nucleotide identity. Antonie Van Leeuwenhoek.

[29] Eid J, Fehr A, Gray J, Luong K, Lyle J, Otto G, Peluso P, Rank D, Baybayan P, Bettman B (2009). Real-Time DNA sequencing from single polymerase molecules. Methods Enzymol.

[30] Vardhan S, Kaushik R, Saxena AK, Arora DK (2011). Restriction analysis and partial sequencing of the 16S rRNA gene as index for rapid identification of *Bacillus* species. Antonie Van Leeuwenhoek.

[31] Zhang M, Li R, Cao L, Shi J, Liu H, Huang Y, Shen Q (2014). Algal sludge from Taihu Lake can be utilized to create novel PGPR-containing bio-organic fertilizers. J Environ Manage.

[32] Gupta R, Bisaria VS, Sharma S (2015). Effect of agricultural amendments on *Cajanus cajan* (Pigeon Pea) and its rhizospheric microbial communities—a comparison between chemical fertilizers and bioinoculants. PLoS ONE.

[33] Afzal M, Shabir G, Tahseen R, Islam EU, Iqbal S, Khan QM, Khalid ZM (2014). Endophytic *Burkholderia* sp. strain PsJN improves plant growth and phytoremediation of soil irrigated with textile effluent. CLEAN.

[34] Naveed M, Mitter B, Reichenauer TG, Wieczorek K, Sessitsch A (2014). Increased drought stress resilience of maize through endophytic colonization by *Burkholderia phytofirmans* PsJN and *Enterobacter* sp. FD17. Environ Exp Bot.

[35] Atkins SD, Clark IM, Pande S, Hirsch PR, Kerry BR (2005). The use of real-time PCR and species-specific primers for the identification and monitoring of *Paecilomyces lilacinus*. Fems Microbiol Ecol.

[36] Haarman M, Knol J (2006). Quantitative real-time PCR analysis of fecal *Lactobacillus* species in infants receiving a prebiotic infant formula. Appl Environ Microbiol.

[37] Ruppel S, Rühlmann J, Merbach W (2006). Quantification and localization of bacteria in plant tissues using quantitative real-time PCR and online emission fingerprinting. Plant Soil.

[38] Couillerot O, Bouffaud ML, Baudoin E, Muller D, Caballero-Mellado J, Moënne-Loccoz Y (2010). Development of a real-time PCR method to quantify the PGPR strain *Azospirillum lipoferum* CRT1 on maize seedlings. Soil Biol Biochem.

[39] Couillerot O, Poirier MA, Prigent-Combaret C, Mavingui P, Caballero-Mellado J, Moënne-Loccoz Y (2010). Assessment of SCAR markers to design real-time PCR primers for rhizosphere quantification of *Azospirillum brasilense* phytostimulatory inoculants of maize. J Appl Microbiol.

[40] Havlak P, Chen R, Durbin KJ, Egan A, Ren Y, Song XZ, Weinstock GM, Gibbs RA (2004). The Atlas genome assembly system. Genome Res.

[41] Stets MI, Alqueres SM, Souza EM, Pedrosa FO, Schmid M, Hartmann A, Cruz LM (2015). Quantification of *Azospirillum brasilense* FP2 bacteria in wheat roots by strain-specific quantitative PCR. Appl Environ Microbiol.

[42] Wang Z, Qian K, Lu JX, Zou LL, Chen D, Sun BC (2009). Realtime quantitative polymerase chain reaction determining *bla CTX-M-14* gene in bacterium. Chin J Antibiot.

[43] Wagg C, Bender SF, Widmer F, Mg VDH (2014). Soil biodiversity and soil community composition determine ecosystem multifunctionality. Proc Natl Acad Sci USA.

[44] Ge B, Liu B, Nwet TT, Zhao W, Shi L, Zhang K (2016). *Bacillus methylotrophicus* strain NKG-1, isolated from Changbai Mountain, China, has potential applications as a biofertilizer or biocontrol agent. PLoS ONE.

[45] Palazzini JM, Dunlap CA, Bowman MJ, Chulze SN (2016). *Bacillus velezensis* RC 218 as a biocontrol agent to reduce fusarium head blight and deoxynivalenol accumulation: genome sequencing and secondary metabolite cluster profiles. Microbiol Res.

[46] Kalam S, Das SN, Basu A, Podile AR (2017). Population densities of indigenous Acidobacteria change in the presence of plant growth promoting rhizobacteria (PGPR) in rhizosphere. J Basic Microbiol.

