# Supplementary figures and images for: Application of Bacillus velezensis NJAU-Z9 Enhanced Plant Growth Associated with Efficient Rhizospheric Colonization Monitored by qPCR with Primers Designed from the Whole Genome Sequence
Source: Curr Microbiol. 2018 Sep 7;75(12):1574–83. doi: 10.1007/s00284-018-1563-4 (PMC6208667; doi:10.1007/s00284-018-1563-4)

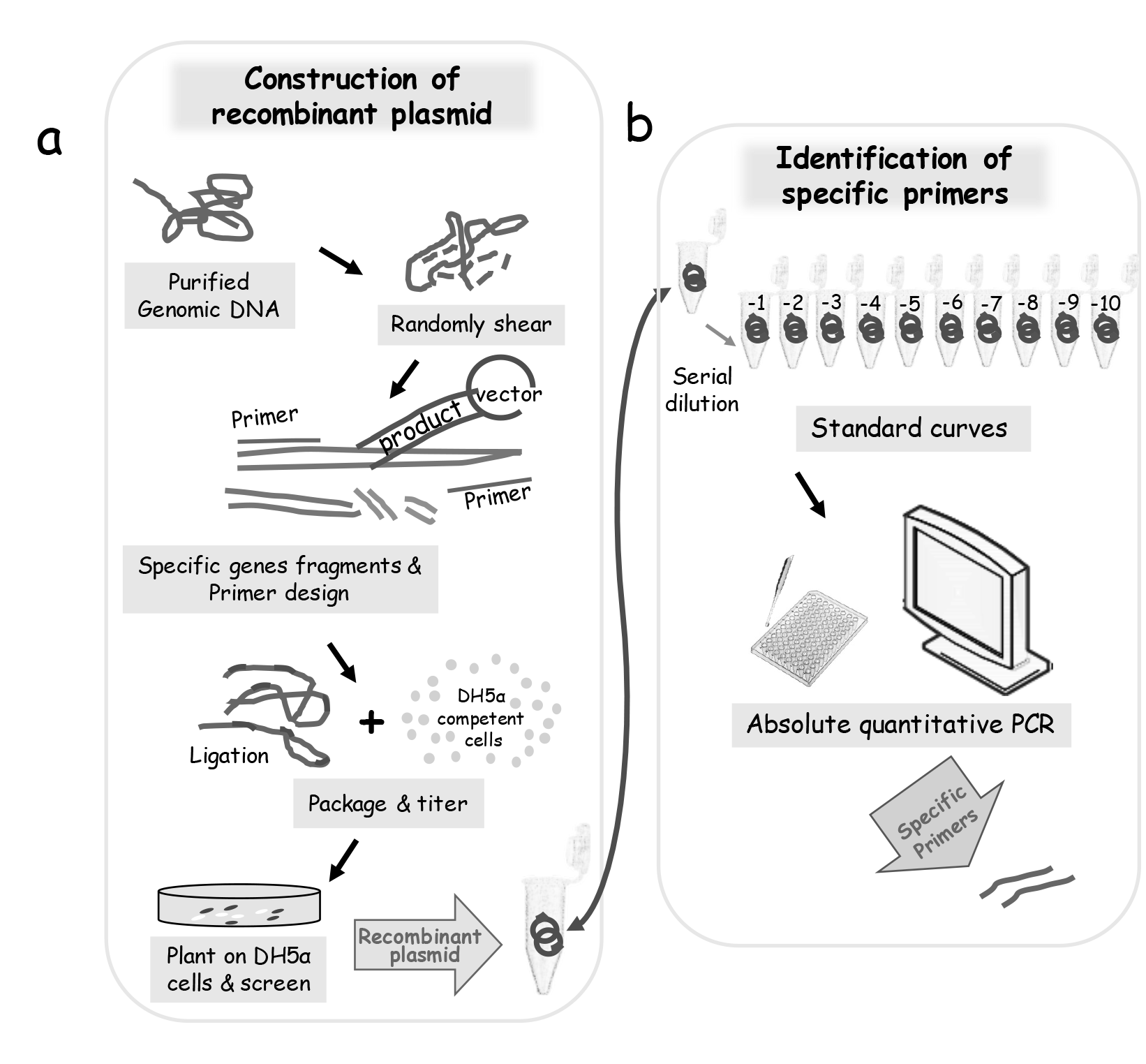

Supplement: Supplementary file 1 — Supplementary material 1 (TIF 2174 KB) [file 284_2018_1563_MOESM1_ESM.tif]

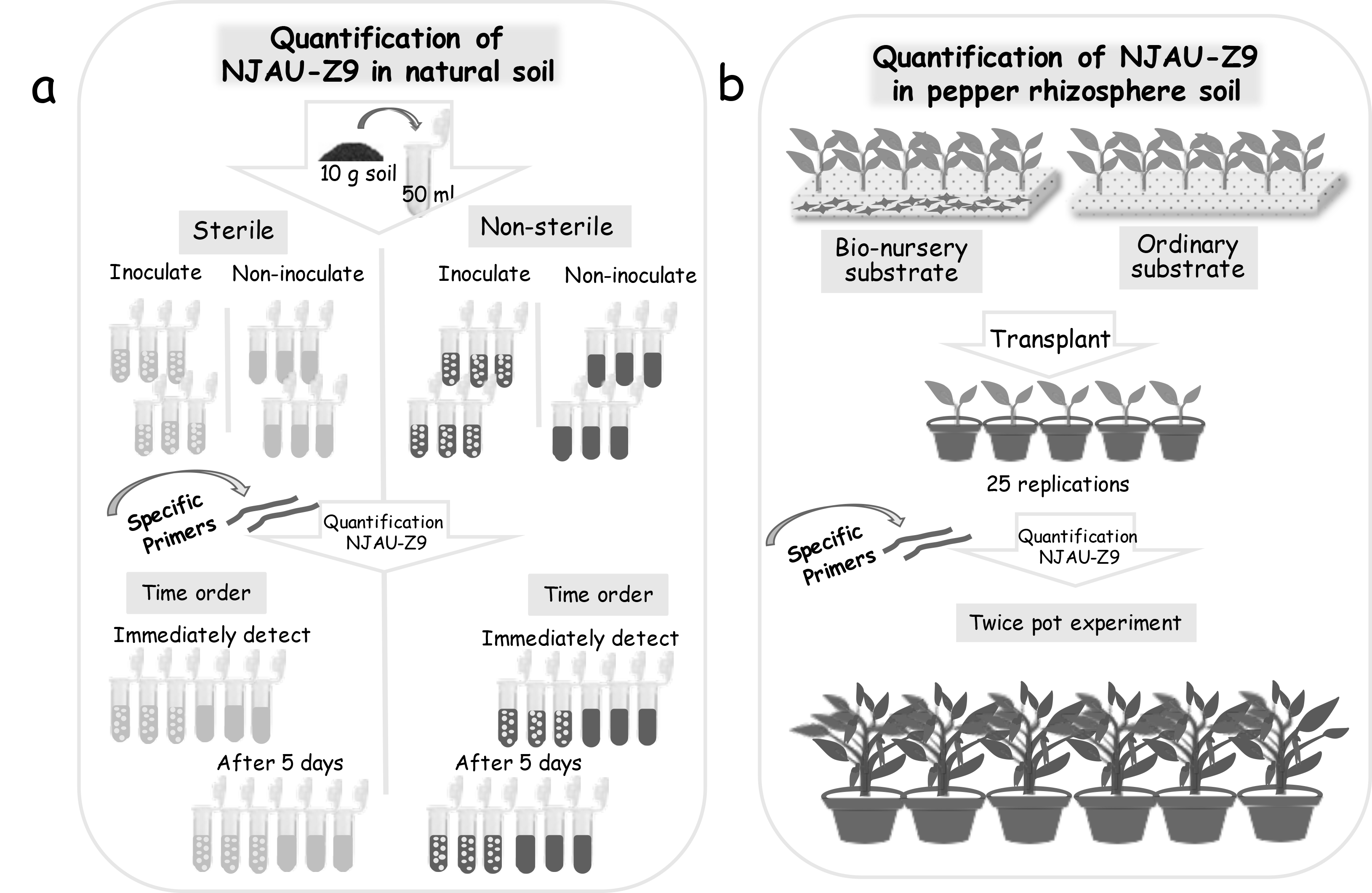

Supplement: Supplementary file 2 — Supplementary material 2 (TIF 1061 KB) [file 284_2018_1563_MOESM2_ESM.tif]

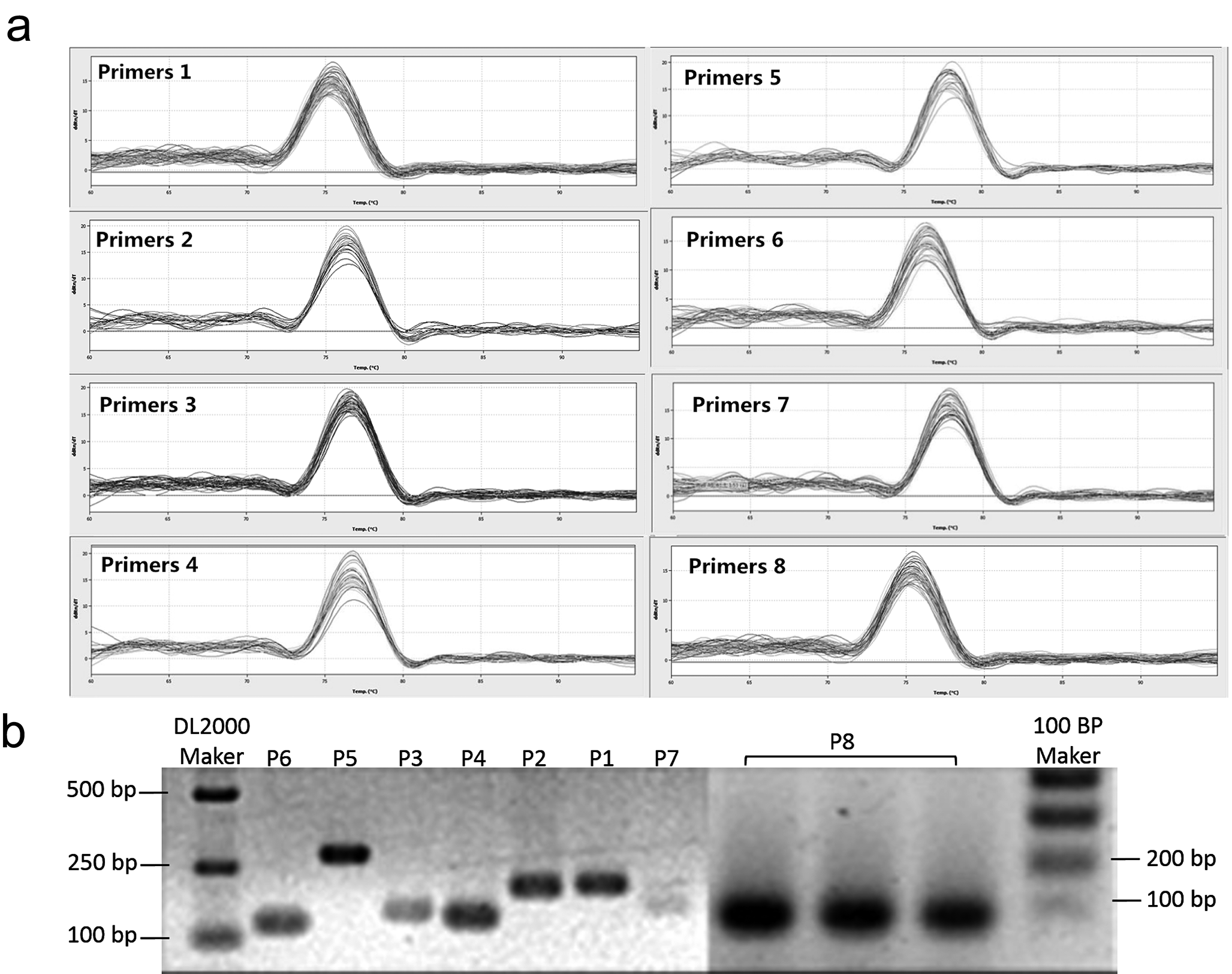

Supplement: Supplementary file 3 — Supplementary material 3 (TIF 1497 KB) [file 284_2018_1563_MOESM3_ESM.tif]

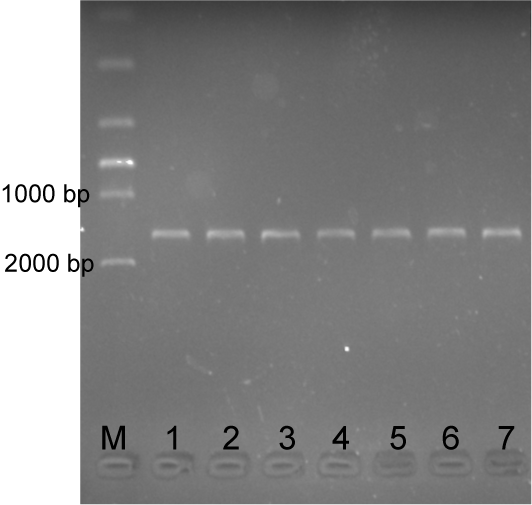

Supplement: Supplementary file 4 — Supplementary material 4 (TIF 795 KB) [file 284_2018_1563_MOESM4_ESM.tif]

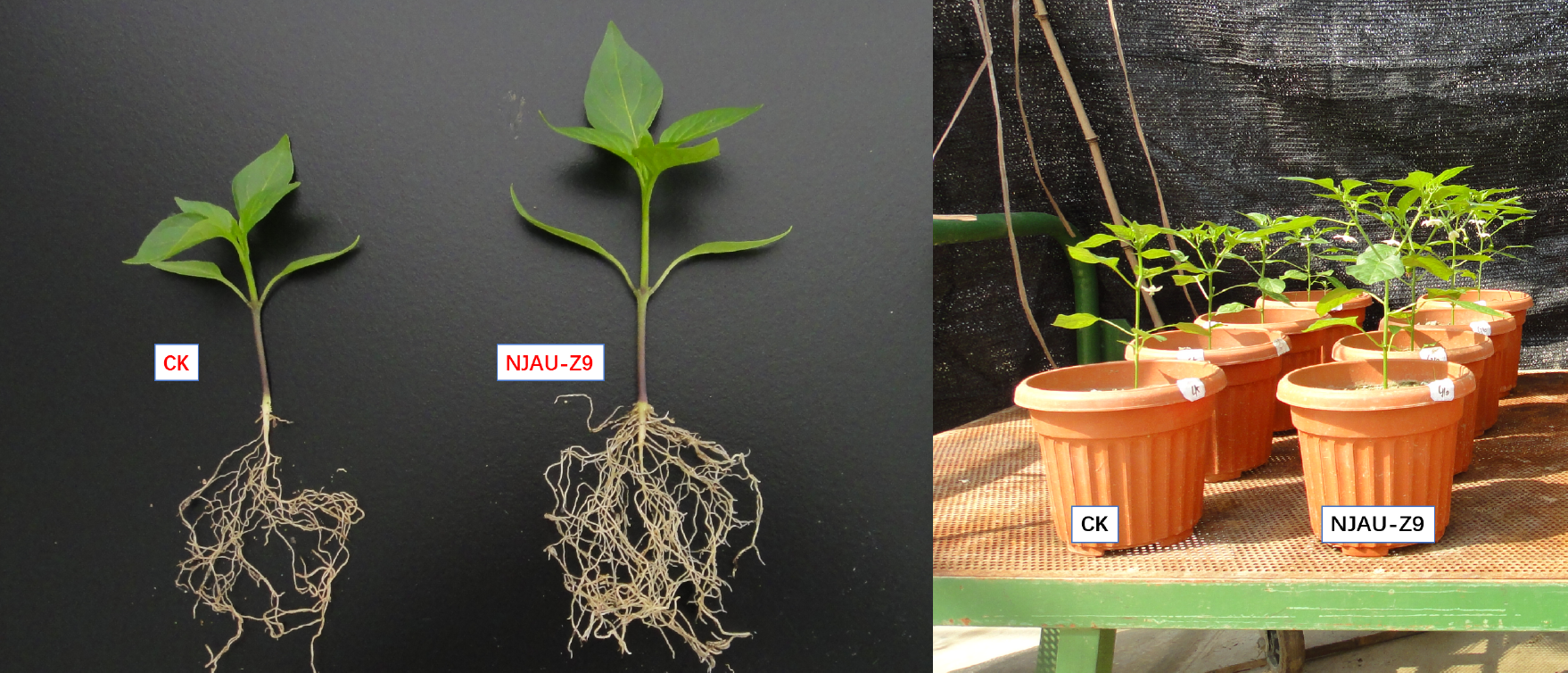

Supplement: Supplementary file 5 — Supplementary material 5 (TIF 2099 KB) [file 284_2018_1563_MOESM5_ESM.tif]

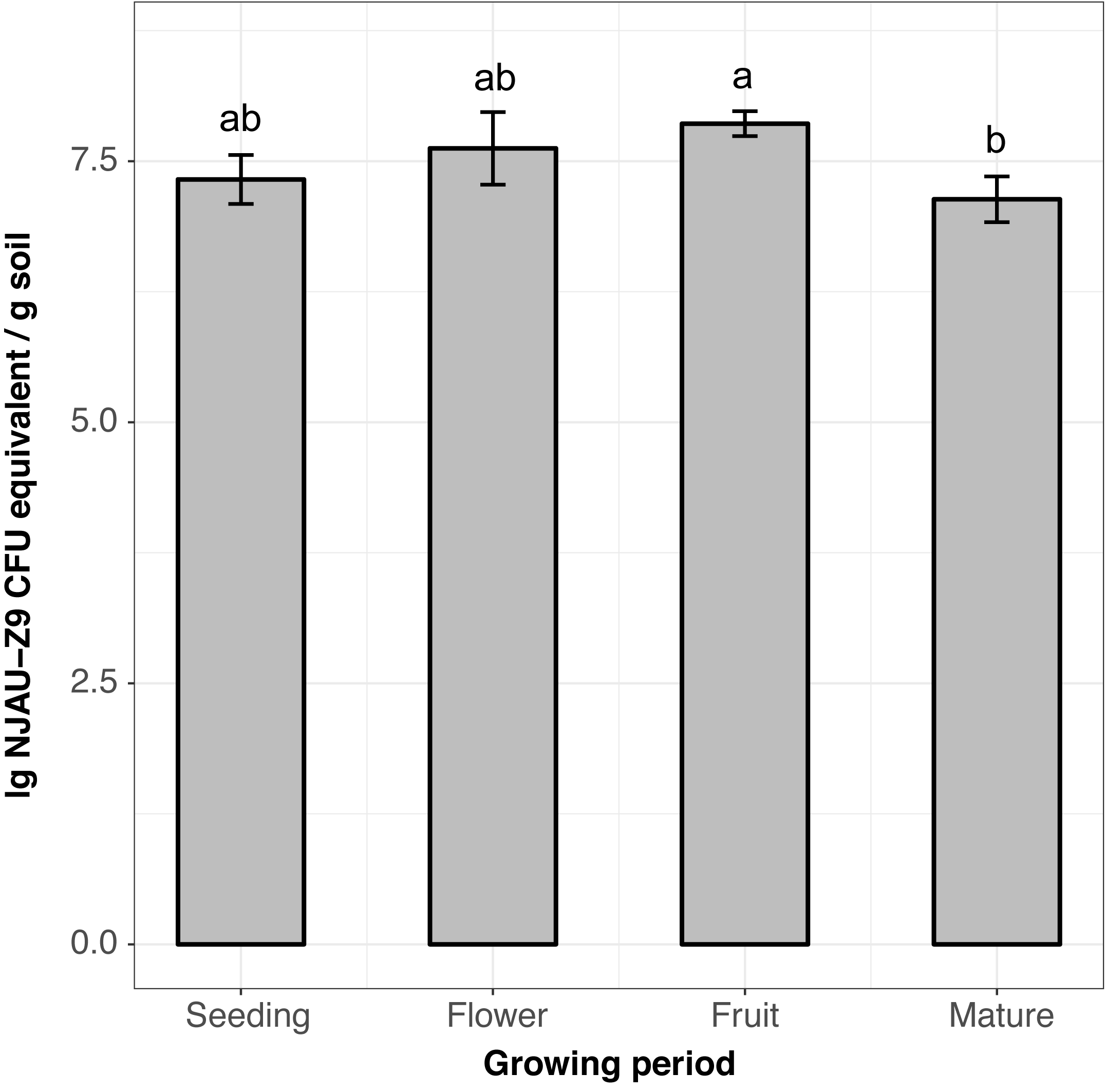

Supplement: Supplementary file 6 — Supplementary material 6 (TIF 1074 KB) [file 284_2018_1563_MOESM6_ESM.tif]
