# Supplementary material for: Application of Bacillus velezensis NJAU-Z9 Enhanced Plant Growth Associated with Efficient Rhizospheric Colonization Monitored by qPCR with Primers Designed from the Whole Genome Sequence
Source: Curr Microbiol. 2018 Sep 7;75(12):1574–83. doi: 10.1007/s00284-018-1563-4 (PMC6208667; doi:10.1007/s00284-018-1563-4)
Supplement: Supplementary file 7 — Supplementary material 7 (DOCX 25 KB) [file 284_2018_1563_MOESM7_ESM.docx]

**Table S1** Genome sequence information of closest type strains and average nucleotide identity with NJAU-Z9

| Strains | Similarity to NJAU-Z9 (ANI, %) | GC conten (%) | Genome size (Mb) | Genomic sequence (Accession NO.) |
| --- | --- | --- | --- | --- |
| *Bacillus methylotrophicus* CBMB205^T^ | 97.94 | 46.78 | 3.7 | NZ_CP011937.1 |
| *Bacillus velezensis* FZB42^T^ | 97.8 | 46.46 | 4 | NC_009725.1 |
| *Bacillus amyloliquefaciens* DSM 7^T^ | 94.07 | 46.08 | 4 | FN597644.1 |

**Table S2** Bacterial strains used in this study

| Microorganism | Accession numbers | Reference |
| --- | --- | --- |
| *Bacillus subtilis* NJAU-G10 | KP403800 | [24] |
| *Bacillus amyloliquefaciens* SQR-9 | CP006890 | [48] |
| *Bacillus amyloliquefaciens* T-5 | JQ217371.1 | [49] |
| *Bacillus pumilus* LZ-8 | GQ850587.1 | [50] |
| *Bacillus amyloliquefaciens* NJN-6 | CP007165 | [51] |
| *Bacillus vallismortis* NJAU-N23 | AB021198 | [52] |

**Table S3** Characteristics of NJAU-Z9 by comprehensive analysis

| Characteristic | NJAU-Z9 | Characteristic | NJAU-Z9 | Characteristic | NJAU-Z9 | Characteristic | NJAU-Z9 |
| --- | --- | --- | --- | --- | --- | --- | --- |
| Pigmentation | Creamy white | D-Mannose | + | D-Raffinose | / | β-Galactosidase | / |
| ammonia | + | D-Fructose | + | α-D-Lactose | / | L-Fucose | — |
| Indole Acetic Acid | + | D-Sorbitol | + | Adipic acid | — | L-Rhamnose | — |
| Antagonism towards pathogens | + | D-Mannitol | + | D-Fucose | — | α-Keto-Butyric Acid | — |
| Dextrin | + | L-Arginine | + | Inosine | — | D-Arabitol | — |
| D-Maltose | + | L-Aspartic Acid | + | α-Keto-Glutaric Acid | — | Anaerobic growth | — |
| D-Ribose | + | L-Glutamic Acid | + | D-Malic Acid | — | pH 6 | + |
| D-Trehalose | + | L-Histidine | + | Tween 40 | — | pH 5 | + |
| D-Cellobiose | + | Ethanol | + | D-Melibiose | / | 10% NaCl | — |
| Gentiobiose | + | Methyl Pyruvate | + | Gelatin | / | 1% NaCl | + |
| Sucrose | + | L-Lactic Acid | + | Glycyl-L-Proline | / | 4% NaCl | + |
| β-Methyl-D Glucoside | + | Methanol | + | L-Alanine | / |  |  |
| L-Malic Acid | + | Citric Acid | + | D-Galactose | / |  |  |
| D-Salicin | + | Valeric acid | — | Sodium acetate | — |  |  |
| α-D-Glucose | + | Urease | — | 3-Methyl Glucose | — |  |  |

Note：+, Growth; —, no growth; /, weak growth.

24. Zhang Y, Wen CY, Zhao MQ, Zhang M, Gao Q, Li R, Shen QR (2015) Isolation of plant growth promoting rhizobacteria from pepper and development of bio-nursery substrates. Journal of Nanjing Agricultural University

48. Zhang N, Yang D, Wang D, Miao Y, Shao J, Zhou X, Xu Z, Li Q, Feng H, Li S (2015) Whole transcriptomic analysis of the plant-beneficial rhizobacterium Bacillus amyloliquefaciens SQR9 during enhanced biofilm formation regulated by maize root exudates. Bmc Genomics 16 (1):685

49. Tan S, Jiang Y, Song S, Huang J, Ling N, Xu Y, Shen Q (2013) Two Bacillus amyloliquefaciens strains isolated using the competitive tomato root enrichment method and their effects on suppressing Ralstonia solanacearum and promoting tomato plant growth. Crop Protection 43 (1):134-140

50. Mei XL, Zhao QY, Tan SY, Xu YC, Shen B, Shen QR (2010) [Screening, identification, and biocontrol effect of antagonistic bacteria against Phytophthora capsici]. Chinese Journal of Applied Ecology 21 (10):2652

51. Zhang N, Yang D, Kendall JRA, Borriss R, Druzhinina IS, Kubicek CP, Shen Q, Zhang R (2016) Comparative Genomic Analysis of Bacillus amyloliquefaciens and Bacillus subtilis Reveals Evolutional Traits for Adaptation to Plant-Associated Habitats. Frontiers in Microbiology 7 (2039). doi:10.3389/fmicb.2016.02039

52. ZHANG Y, WANG T, SUN Y, HU G, LI R, YU P, Qirong S (2017) Screening of Plant Growth-Promoting Rhizobacteria from Watermelon and Development of Bio-nursery Substrates. ACTA PEDOLOGICA SINICA 54 (3):702-712. doi:10.11766/trxb201608300287
